# Composite dietary antioxidant index and sleep health: a new insight from cross-sectional study

**DOI:** 10.1186/s12889-024-18047-2

**Published:** 2024-02-26

**Authors:** Bingquan Xiong, Jiaxin Wang, Rui He, Guangsu Qu

**Affiliations:** 1 Department of Cardiology, The Second Affilliated Hospital of Chongqing Medical University, No.74, Linjiang Road, Chonqing, 400010 China; 2 Department of Endocrinology and Metabolism, The First Affiliated Hospital of Jinan University, No.613, Huangpu Road, Guangzhou, Guangdong Province 510632 China; 3 Department of Respiratory and Critical Care Medicine, The Second Affilliated Hospital of Chongqing Medical University, No.74, Linjiang Road, Chonqing, 400010 China

**Keywords:** Sleep, Dietary, Antioxidant, Nutrition, Oxidative stress, National Health and Nutrition Examination Survey

## Abstract

**Background:**

Low-quality sleep and obstructive sleep apnea (OSA) can result in series of chronic diseases. Healthy diet has been considered as an effective and simple strategy to optimize sleep quality. However, current evidence on the correlation of dietary composite antioxidant intake with sleep health remained obscure.

**Aim of the study:**

To determine the relationship of composite dietary antioxidant index (CDAI) and sleep health.

**Methods:**

Cross-sectional analyses were based on National Health and Nutrition Examination Survey (NHANES) 2005–2008. Dietary consumption was assessed by trained staff using 24-h diet recall method and CDAI was calculated based on previous validated approach that included six antioxidants. Sleep-related outcomes were self-reported by a set of questionnaires and classified into OSA, day sleepiness, and insufficient sleep. Weighted logistic regression was conducted to calculate odds ratios (ORs) and 95% confidence intervals (CIs). Restricted cubic spline (RCS) regressions were also used to evaluate the dose-response of CDAI and three sleep-related outcomes.

**Results:**

A total of 7274 subjects included (mean age: 46.97 years) were enrolled in our study, including 3658 were females (52.54%) and 3616 were males (47.46%). Of them, 70.6%, 29.51%, and 35.57% of the subjects reported that they had OSA, day sleepiness and insufficient sleep, respectively. Logistic regression showed the highest quartile of CDAI was inversely associated with the risk of OSA (OR: 0.69, 95%CI: 0.49–0.97), day sleepiness (OR: 0.64, 95%CI: 0.44–0.94) and insufficient sleep (OR: 0.68, 95%CI: 0.50–0.92) compared with the lowest quartile. RCS showed linear relationship of CDAI and insufficient sleep but non-linear relationship of CDAI with OSA and day sleepiness.

**Conclusions:**

Our results show that CDAI was non-linearly associated with lower risk of OSA and day sleepiness whereas a linear inverse association between CDAI and insufficient sleep was observed. These findings implicate that combined intake of antioxidants could be a promising and effective approach to optimize sleep quality for public.

## Introduction

Over the past decades, sleep disorders have been confirmed a common concern that affected 50–70 million U.S. population [1] and brought serious threat to public health worldwide. Low-quality sleep including obstructive sleep apnea (OSA) and insufficient sleep was highly related to a series of adverse outcomes, including cancer, diabetes, metabolic syndrome and cardiovascular diseases [2]. Although continuous positive airway pressure (CPAP), reducing excessive drowsiness and improve patients’ quality of life, is the recommendable and standard treatment for OSA [3], the adherence rate of CPAP is only ranging from 30 to 60%, which significantly affects the therapeutic outcomes of OSA patients [4]. Therefore, effective early prevention, including the development of a healthy lifestyle and diet, is currently an urgent need for the prevention of sleep disorders.

The imbalance of prooxidants and antioxidants of diet could contribute to sleep disorders [5–8]. However, little epidemiological evidence has focused on the total antioxidant property of diet in the pathogenesis of sleep disorders. A possible explanation was that researchers did not take into account the potential interaction and synergistic effects between different antioxidants [9]. In fact, literatures of individual antioxidant nutrient on OSA are scarce and the results are not consistent. More importantly, a previous meta-analysis concluded that an increase in antioxidant supplement was not always beneficial and sometimes acted as cocarcinogen to increase mortality [10]. Conversely, intake of nutrients from foods and beverages might bring more positive influence on our body because they were rich in flavones, dietary fiber, phenolic compounds, folic acid and other antioxidants. Recently, composite dietary antioxidant index (CDAI), consisted of vitamin A, C, E, selenium, zinc and carotenoids, has been considered as a comprehensive and promising index to assess the cumulative effect of total dietary antioxidant capacity (DTAC). However, previous studies investigating the relationship between sleep disorders and DTAC mostly had limited study simple size and focused on specific population, such as diabetic women [11] and post-menopausal women [12].

To our knowledge, no large-scale investigations have explored whether DTAC could reduce the risk of OSA and other sleep disorders among overall population. To fill in these knowledge gaps, we obtained public data from the National Health and Nutrition Examination Survey (NHANES) to assess the effect of CDAI on sleep disorders among Americans, including OSA, day sleepiness and insufficient sleep.

## Methods

### Study design

The NHANES, a periodic and nationally program with a series of multistage stratified sample design, conducted by the National Center for Health Statistics (NCHS) to survey the nutritional and health status of US adults [13]. The NCHS signed all informed consent which was obtained from each participants at the time of recruitment [14]. Detailed information regarding the survey design, codebooks, and methodologies employed in NHANES can be accessed on the official NHANES website: https://www.cdc.gov/nchs/nhanes/index.htm.

**Fig. 1:**
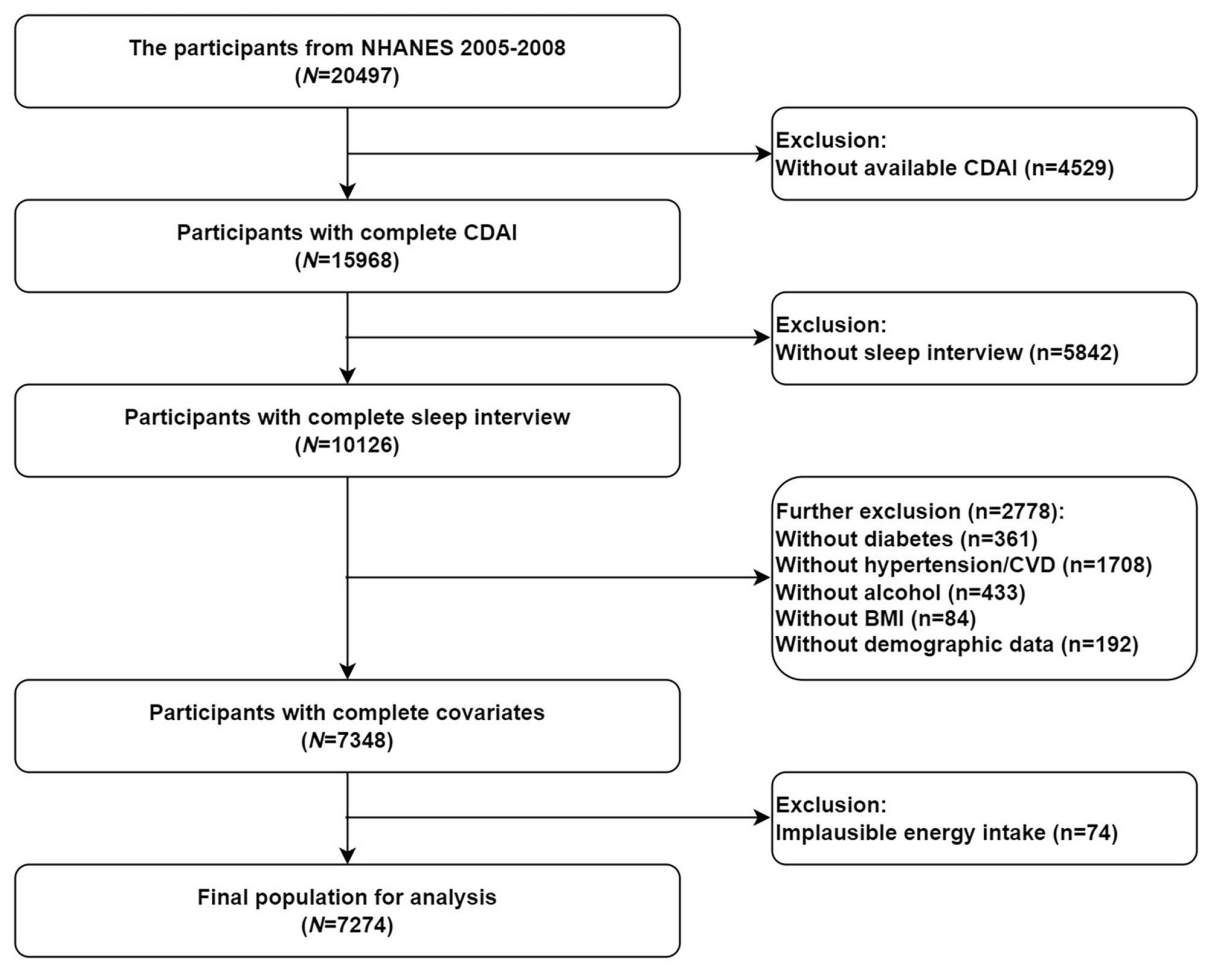
Flow chart of the study

The current research analyzed data focused on the NHANES 2005–2006 and 2007–2008 cycles. Specifically, a total of 20,497 individuals from these two continuous cycles were surveyed in current investigation. Among them, we excluded those without available CDAI data (*n* = 4529), those without sleep interview (*n* = 5842), those who had missing information on potential covariates (*n* = 2778) and further excluded those who had implausible daily energy intake (< 500 kcal per day or > 5000 kcal per day, *n* = 74). Finally, 7274 subjects with complete data were enrolled in our statistical analysis. Figure 1 shows the participants selection flowchart.

### Sleep-related outcomes assessment

A set of complete questionnaires about sleep were publicly provided in NHANES 2005–2008. These questionnaires were consistent with Sleep Heart Health Study. Additionally, the Computer-Assisted Personal Interview (CAPI) was equipped with built-in consistency verification to reduce data entry errors when the trained collected the information about sleep [15]. According to our definition, we choose three self-reported outcomes related to sleep quality in our study. Detailed information about the sleep-related health definition was described as follows.

Sleep duration: In NHANES 2005–2008, sleep duration was determined by the interview “How much sleep do you get(hours)?” and responses were recorded as hours. According to previous literature [16], sleep duration was grouped into insufficient sleep (< 7 h/night), mid-range sleep (7-9 h/night) and excessive sleep (> 9 h/night) [16, 17].

OSA: The diagnosis of OSA was based on one or more of the following four conditions:1) Snoring 3 or more nights/week; 2) snorting, gasping, or stopping breathing 3 or more nights/week; 3) feeling excessively sleepy during the day 16–30 times/month despite sleeping around 7 or more hours/night on weekdays or work nights; or 4) doctor diagnosed sleep apnea [18].

Day sleepiness: In our selective NHANES cycles, day sleepiness was evaluated by following 2 questions that asked, “In the past month, how often did you feel unrested during the day, no matter how many hours of sleep you had?” Another question was “In the past month, how often did you feel excessively or overly sleepy during the day?” The response classifications were “Never”, “Rarely (1 time per month)”, “Sometimes (2–4 times per month)”, “Often (5–15 times per month)” and “Almost always (16–30 times per month)”. Subjects who answered “Often” and “Almost always” were regarded as having day sleepiness [18].

### Composite dietary antioxidant index assessment

The information on dietary and their components was collected by highly trained staffs in NHANES. All participants had two 24-h dietary recall surveys. All NHANES examinees were eligible to take part in these recall interviews. The first 24-hour recall record was determined face-to-face by trained food recall data collectors, then the second was performed over the phone 3–10 days later [19]. In current research, information about dietary intake data were complete for all participants. Hence, the average antioxidant intake over 2 days was used in final statistical analysis to reduce bias. The calculation of CDAI was based on six kinds of antioxidant components, including vitamin A, vitamin C, vitamin E, zinc, selenium, and carotenoids. Specifically, each antioxidant was standardized by subtracting the total mean and dividing the total standard deviation. Next, we summed the standardized intake of individual nutrient to obtain the CDAI according to the equation reported in previous study [20].

### Covariates

According to prior studies and clinical experience, we considered appropriate covariates that associated with sleep quality. Information about age, gender, race, education, marital status, poverty income ratio (PIR) was collected from demographic questionnaires. Obesity was defined as BMI ≥ 30Kg/m^2^. In current study, the diagnosis of hypertension was based on one or more of the following three conditions: (1) Participants answered the blood pressure questionnaire “Has a doctor or other health professional ever told you that you had hypertension?” (2) Participants were regarded as hypertensive if the average systolic blood pressure (SBP) was ≥ 140mmHg or the average diastolic blood pressure (DBP) ≥ 90mmHg. (3) If individuals currently taken any antihypertensive drug, they were also considered as hypertension. Based on prior literature’s definition of alcohol consumption, we categorized these individuals into following four groups:1) Never drinking: no history of alcohol consumption, 2) Current heavy drinking [≥ 3 drinks/day for women, ≥ 4 drinks/day for men, or binge 5 drinks/day or more], 3)current moderate drinking [≥ 2 drinks/day for women, ≥ 3 drinks/day for men, or binge ≥ 2 days/month], 4)current light drinking [≤ 1 drinks/day for women, ≤ 2 drinks/day for men, or had a history of binge drinking] [21]. Diabetes was defined as the definite diagnosis told by doctors or current use of diabetes medications or insulin.

### Statistical analysis

In current analysis, all statistical tests were bilateral, and *p* < 0.050 was defined as statistical significance. All missing values were excluded and R software was conducted for data analysis. To account for the complexity of sampling design in NHANES 2005–2006 and 2007–2008, the 2-year sample weights were applied in our final analysis according to the stipulated analytical guidelines [22]. Continuous variables are displayed as mean ± standard error (SE) and categories variables are indicated as frequency with percentage. In order to compare the difference among baseline characteristics by CDAI quartile groups, we used one-way ANOVA tests for continuous variables and chi-square tests for categorical variables. The intakes of dietary factors were further adjusted for total energy intake using the residual method [23]. A series of logistic regressions were built to estimate the odds ratios (ORs) and 95% confidence intervals (CIs) between CDAI and different sleep-related outcomes after adjusting confounding factors. Model 1 was a crude model. Model 2 adjusted for age, gender, race and marital status. Model 3 was additionally adjusted all covariates in model 2 plus education level, PIR, smoke status, alcohol status, cardiovascular disease (CVD), hypertension, diabetes and daily intake of energy, caffeine, carbohydrate, fat, protein, and fiber. Additionally, we performed CDAI as a continuous variable in these logistic models. Of note, we also examined the ORs (95% CIs) per 1-standard deviation (SD) increase in different models. The risk of sleep-related outcomes and daily intake of individual antioxidant were also evaluated in the fully-adjusted model. The linear trend was conducted by assigning CDAI quartiles as an ordinal variable in these models. To further visualize relationship between CDAI and each sleep-related outcome, restricted cubic spline (RCS) was also conducted in our analysis. Finally, subgroup analyses were used to investigate the correlation between CDAI and each sleep-related outcome in different population. Likelihood ratio test was conducted to calculate interaction among these subgroups.

## Results

### Baseline characteristics of participants

The sample size from NHANES 2005–2008 was 7274, representing a total population-based size of 96,752,239 females and 87,400,859 males in the United States. Characteristics of subjects according to CDAI quartiles (Q1: CDAI ≤-2.30; Q2: -2.30 < CDAI≤-0.19; Q3: -0.19 < CDAI ≤ 2.49; Q4: CDAI > 2.49) are illustrated in Table 1. Of the 7274 participants (mean age: 46.97 ± 0.47 years), 3616 (47.46%) were males and 3658 (52.54%) were females in this analysis. In addition, most participants were overweight with the mean BMI of the subjects was approximately 28.70Kg/m^2^. In addition, approximately 70.06%, 29.51%, and 35.57% of the participants reported that they had OSA, day sleepiness and insufficient sleep, respectively. Compared with the lowest quartile (Q1), the subjects in the highest CDAI quartile (Q4) were more likely to be younger adults, male, married, mild alcoholics, non-Hispanic White, college-educated, have higher intake of energy, carbohydrate, fat, protein, fiber, and caffeine, and have a PIR > 3.50 while were less prone to be smokers and have lower prevalence of diabetes and CVD (all *p* < 0.050). No significant difference was observed among four CDAI categories on hypertension and BMI (all *p* > 0.050).

**Table 1:** Characteristics of participants stratified by the CDAI, weighted (*n* = 7274)^a^

Variables	Total(*n* = 7274)	Q1(*n* = 1823)	Q2(*n* = 1815)	Q3(*n* = 1817)	Q4(*n* = 1819)	*P*
Age,years	46.97 ± 0.47	47.12 ± 0.48	48.01 ± 0.66	46.86 ± 0.72	46.09 ± 0.62	0.035
BMI,kg/m^2^	28.70 ± 0.19	28.75 ± 0.26	28.92 ± 0.18	28.71 ± 0.28	28.46 ± 0.30	0.328
Energy,kcal/d	2093.55 ± 18.58	1447.62 ± 24.14	1871.36 ± 21.21	2215.35 ± 18.65	2657.78 ± 24.75	< 0.001
Carbohydrate,g/day	252.80 ± 2.16	181.14 ± 3.69	226.99 ± 2.76	266.53 ± 3.26	316.12 ± 2.22	< 0.001
Fat,g/day	79.79 ± 0.95	52.95 ± 0.93	70.74 ± 0.86	85.26 ± 1.09	102.72 ± 1.45	< 0.001
Protein,g/day	82.53 ± 0.82	52.06 ± 0.87	72.62 ± 0.77	86.91 ± 0.71	109.94 ± 1.49	< 0.001
Fiber,g/day	15.99 ± 0.28	9.26 ± 0.18	13.44 ± 0.18	16.77 ± 0.19	22.49 ± 0.39	< 0.001
Caffeine,g/day	179.23 ± 4.88	183.59 ± 11.88	176.65 ± 7.39	174.23 ± 7.19	182.58 ± 7.79	0.887
Gender,n(%)						< 0.001
Female	3658(52.54)	1010(59.94)	909(52.75)	875(49.64)	864(49.35)	
Male	3616(47.46)	813(40.06)	906(47.25)	942(50.36)	955(50.65)	
Race,n(%)						0.001
Mexican American	1229(7.54)	336(8.17)	307(8.05)	309(8.03)	277(6.20)	
Non-Hispanic Black	1523(10.61)	460(13.83)	388(11.61)	358(10.15)	317(7.74)	
Non-Hispanic White	3775(73.58)	824(69.19)	927(71.41)	965(74.12)	1059(78.22)	
Other races	747(8.27)	203(8.81)	193(8.93)	185(7.70)	166(7.83)	
Education,n(%)						< 0.001
<High school	1965(17.30)	689(26.64)	513(17.71)	419(14.97)	344(11.96)	
High school	1781(25.17)	497(30.37)	480(28.10)	410(23.76)	394(20.10)	
>High school	3528(57.52)	637(42.98)	822(54.19)	988(61.27)	1081(67.95)	
Marital status,n(%)						0.001
Unmarried	1075(16.52)	298(19.16)	261(16.53)	241(14.55)	275(16.30)	
Married	4036(56.90)	919(50.06)	1002(56.11)	1078(60.14)	1037(59.82)	
Others	2163(26.57)	606(30.78)	552(27.36)	498(25.30)	507(23.88)	
PIR,n(%)						< 0.001
≤ 1.30	1950(18.16)	643(25.47)	515(20.78)	415(14.51)	377(13.77)	
1.30–3.50	2867(36.00)	766(40.40)	732(37.26)	713(35.26)	656(32.29)	
> 3.50	2457(45.83)	414(34.13)	568(41.96)	689(50.23)	786(53.93)	
Hypertension,n(%)	3140(37.11)	873(39.90)	810(38.75)	745(35.63)	712(34.99)	0.149
Diabetes,n(%)	910(8.47)	264(8.67)	252(10.94)	208(8.06)	186(6.68)	0.005
Smoke,n(%)	1556(23.13)	527(34.36)	376(23.14)	356(21.09)	297(16.38)	< 0.001
CVD,n(%)	850(8.33)	278(11.46)	226(9.53)	190(8.01)	156(5.24)	< 0.001
Alcohol status,n(%)						< 0.001
Never	2564(28.43)	788(36.00)	682(30.73)	565(25.30)	529(23.63)	
Mild	2300(34.68)	436(25.69)	555(32.07)	640(36.70)	669(41.86)	
Moderate	1063(16.28)	255(14.59)	240(15.38)	275(17.65)	293(17.04)	
Heavy	1347(20.61)	344(23.72)	338(21.82)	337(20.35)	328(17.47)	
OSA,n(%)	5019(70.06)	1248(72.86)	1259(70.07)	1238(69.46)	1274(68.44)	0.184
Day sleepiness,n(%)	1953(29.51)	501(34.12)	461(27.85)	497(30.06)	494(26.85)	0.035
Insufficient Sleep,n(%)	2809(35.57)	770(40.63)	728(38.47)	684(34.94)	627(29.91)	< 0.001

Continuous variables were showed as mean ± SE,categorical variables were showed as frequency (percentage)

Q1:CDAI≤-2.30; Q2:-2.30 < CDAI≤-0.19; Q3:-0.19 < CDAI ≤ 2.49; Q4:CDAI > 2.49.

^a^All estimates accounted for complex survey designs, and all percentages were weighted

Abbreviations:, quartile; SE, standard error; CDAI, composite dietary antioxidant index; BMI, body mass index; PIR, poverty income ratio; CVD, cardiovascular disease; OSA, Obstructive sleep apnea

### Association between CDAI and sleep health

**Fig. 2:**
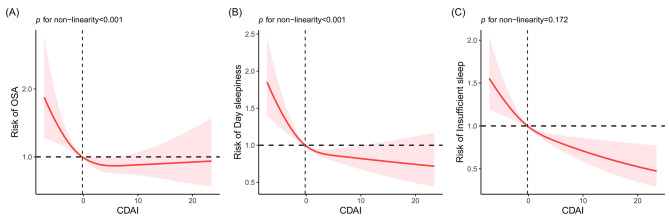
Restricted cubic spline (RCS) analysis with multivariate-adjusted associations between CDAI and three sleep-related outcomes

The associations between CDAI and three sleep-related outcomes are illustrated in Table 2. Compared with the lowest quartile (Q1), an inverse association between the highest quartile (Q4) and each sleep-related outcome was observed in fully-adjusted model. After adjusting all potential confounders, the OR (95%CI) comparing participants in Q4 vs. Q1 of CDAI was 0.69 (0.49, 0.97) for OSA, 0.64 (0.44, 0.94) for day sleepiness, and 0.68 (0.50, 0.92) for insufficient sleep (all *p* < 0.050). What’s more, we converted CDAI from a categorical exposure to a continuous exposure, a similar negative association was observed for CDAI with each sleep-related outcome in fully adjusted model (OSA: β = 0.98, 95%CI: 0.95-1.00; day sleepiness: β = 0.97, 95%CI: 0.94–0.99; insufficient sleep: β = 0.96, 95%CI: 0.93–0.99).The results of ORs (95%CIs) per 1-SD increase supported above findings in the same model (OSA: OR = 0.92, 95%CI: 0.80–0.99; day sleepiness: OR = 0.88, 95%CI: 0.79–0.98; insufficient sleep: OR = 0.85, 95%CI: 0.76–0.96). Additionally, RCS displayed non-linear relationship between CDAI and the risk of OSA (p for non-linearity < 0.001) (Fig. 2**(A)**) and day sleepiness (p for non-linearity < 0.001) (Fig. 2**(B)**), suggesting plateaued associations at the higher CDAI exposure range. However, linear inverse correlation was found between CDAI and insufficient sleep risk (p for non-linearity = 0.172) (Fig. 2**(C)**).

**Table 2:** Weighted logistic regression analyses of CDAI with three sleep-related outcomes

Variables	Model 1		Model 2		Model 3	
	OR (95%CI)	*P*	OR (95%CI)	*P*	OR (95%CI)	*P*
**OSA**						
CDAI	0.99(0.97,1.00)	0.063	0.98(0.97,1.00)	0.018	0.98(0.95,1.00)	0.041
CDAI						
Q1	Reference		Reference		Reference	
Q2	0.87(0.72,1.06)	0.170	0.84(0.68,1.03)	0.091	0.81(0.63,1.05)	0.101
Q3	0.85(0.69,1.04)	0.113	0.79(0.64,0.98)	0.033	0.74(0.54,1.01)	0.058
Q4	0.81(0.66,0.98)	0.042	0.75(0.60,0.94)	0.014	0.69(0.49,0.97)	0.035
*P* for trend		0.041		0.012		0.029
Per 1-SD increase	0.94(0.89,1.00)	0.063	0.92(0.87,0.98)	0.018	0.92(0.80,0.99)	0.041
**Day sleepiness**						
CDAI	0.98(0.95,1.01)	0.111	0.98(0.95,1.00)	0.078	0.97(0.94,0.99)	0.027
CDAI						
Q1	Reference		Reference		Reference	
Q2	0.75(0.57,0.97)	0.028	0.78(0.60,1.01)	0.060	0.75(0.54,1.03)	0.070
Q3	0.83(0.64,1.08)	0.163	0.86(0.66,1.13)	0.274	0.81(0.59,1.12)	0.164
Q4	0.71(0.51,0.98)	0.038	0.72(0.52,0.98)	0.036	0.64(0.44,0.94)	0.029
*P* for trend		0.075		0.068		0.034
Per 1-SD increase	0.91(0.82,1.02)	0.111	0.91(0.82,1.01)	0.078	0.88(0.79,0.98)	0.027
**Insufficient Sleep**						
CDAI	0.96(0.94,0.97)	< 0.001	0.96(0.94,0.98)	< 0.001	0.96(0.93,0.99)	0.013
CDAI						
Q1	Reference		Reference		Reference	
Q2	0.91(0.77,1.09)	0.292	0.92(0.77,1.10)	0.330	0.95(0.75,1.20)	0.609
Q3	0.78(0.65,0.95)	0.013	0.79(0.66,0.96)	0.020	0.82(0.64,1.07)	0.121
Q4	0.62(0.51,0.76)	< 0.001	0.64(0.52,0.79)	< 0.001	0.68(0.50,0.92)	0.021
*P* for trend		< 0.001		< 0.001		0.014
Per 1-SD increase	0.83(0.77,0.90)	< 0.001	0.84(0.78,0.92)	< 0.001	0.85(0.76,0.96)	0.013

Model 1: None

Model 2: Age, gender, race, marital status;

Model 3: Age, gender, race, marital status, education, PIR, BMI, smoke, alcohol status, CVD, hypertension, diabetes status, energy intakes, caffeine, carbohydrate, fat, protein, and fiber

Abbreviations: OSA, Obstructive sleep apnea; CDAI, composite dietary antioxidant index; PIR, poverty income ratio; BMI, body mass index; CVD, cardiovascular disease; OR, odds ratio; CI, confidence interval; Q, quartile; SD, standard deviation

### Individual antioxidant component and sleep-related outcomes

A correlation between specific antioxidant constituent and each sleep-related outcome was assessed by dividing the value of individual antioxidant into quartiles, with the first quartile (Q1) selected as the reference category. Detailed results are showed in Table 3. For OSA, after adjusting all confounding factors, the OR (95%CI) comparing the highest quartile (Q4) with the first quartile (Q1) was 0.69 (0.50–0.96, p for trend = 0.029) for vitamin A, 0.75 (0.57–0.97, p for trend = 0.035) for vitamin C and 0.81 (0.65–0.98, p for trend = 0.036) for carotenoids, respectively. However, these inverse correlations were only found in vitamin E and vitamin C on day sleepiness risk. When comparing with lowest quartile (Q1), the highest quartile (Q4) of vitamin E and vitamin C were both associated with lower day sleepiness in the same fully-adjusted model with the corresponding ORs (95%CIs) were 0.70 (0.53–0.92, p for trend = 0.012) and 0.64 (0.49–0.84, p for trend = 0.020). In addition, we found vitamin A, selenium and carotenoids in the highest quartile (Q4) comparing with the lowest quartile (Q1) were all associated with lower insufficient sleep with the corresponding OR (95%CI) was 0.72 (0.52–0.99, p for trend = 0.036), 0.78 (0.62–0.96, p for trend = 0.035) and 0.77 (0.60–0.98, p for trend = 0.034), respectively. By contrast, these negative associations were not showed in other antioxidants.

**Table 3:** *Multivariable-adjusted logistic regressions for three sleep outcomes according to daily intakes of individual antioxidant components

Variables	OSA		Day sleepiness		Insufficient Sleep	
	OR (95%CI)	*P*	OR (95%CI)	*P*	OR (95%CI)	*P*
Vitamin A,mcg/day						
Q1	Reference		Reference		Reference	
Q2	0.77(0.58,1.03)	0.069	0.93(0.73,1.19)	0.497	0.82(0.63,1.06)	0.114
Q3	0.71(0.54,0.94)	0.023	0.92(0.70,1.23)	0.535	0.75(0.58,0.98)	0.036
Q4	0.69(0.50,0.96)	0.034	0.89(0.66,1.21)	0.402	0.72(0.52,0.99)	0.041
*P* for trend		0.029		0.450		0.036
Vitamin E,mg/day						
Q1	Reference		Reference		Reference	
Q2	1.02(0.76,1.38)	0.865	0.80(0.61,1.04)	0.088	0.97(0.75,1.26)	0.795
Q3	1.03(0.76,1.40)	0.825	0.79(0.62,1.01)	0.055	0.91(0.71,1.16)	0.378
Q4	1.08(0.81,1.42)	0.556	0.70(0.53,0.92)	0.018	0.86(0.68,1.09)	0.169
*P* for trend		0.562		0.012		0.122
Vitamin C,mg/day						
Q1	Reference		Reference		Reference	
Q2	0.85(0.62,1.17)	0.277	0.80(0.60,1.05)	0.096	0.86(0.62,1.19)	0.304
Q3	0.78(0.58,1.05)	0.088	0.78(0.60,1.02)	0.066	0.84(0.63,1.10)	0.172
Q4	0.75(0.57,0.97)	0.035	0.64(0.49,0.84)	0.006	0.85(0.65,1.10)	0.170
*P* for trend		0.020		0.020		0.119
ZinC,mg/day						
Q1	Reference		Reference		Reference	
Q2	0.95(0.71,1.27)	0.686	0.91(0.67,1.23)	0.479	0.92(0.74,1.22)	0.524
Q3	0.82(0.61,1.10)	0.160	0.92(0.70,1.21)	0.494	0.78(0.60,0.99)	0.047
Q4	0.87(0.61,1.24)	0.373	0.89(0.61,1.30)	0.485	0.80(0.69,1.3)	0.281
*P* for trend		0.244		0.520		0.300
Selenium,mcg/day						
Q1	Reference		Reference		Reference	
Q2	0.95(0.74,1.22)	0.641	0.98(0.71,1.34)	0.859	0.72(0.57,0.94)	0.023
Q3	0.95(0.65,1.37)	0.730	0.78(0.55,1.12)	0.147	0.75(0.60,0.95)	0.028
Q4	1.01(0.64,1.59)	0.963	0.96(0.56,1.64)	0.853	0.78(0.62,0.96)	0.034
*P* for trend		0.480		0.472		0.035
Carotenoids,mcg/day						
Q1	Reference		Reference		Reference	
Q2	0.91(0.72,1.15)	0.370	0.84(0.63,1.12)	0.196	0.87(0.73,1.04)	0.100
Q3	0.90(0.69,1.17)	0.386	0.84(0.67,1.05)	0.107	0.93(0.75,1.16)	0.463
Q4	0.81(0.65,0.98)	0.036	0.81(0.63,1.05)	0.101	0.77(0.60,0.98)	0.034
*P* for trend		0.036		0.102		0.042

*Adjusted for age, gender, race, marital status, education, PIR, BMI, smoke, alcohol status, CVD, hypertension, diabetes status, energy intakes, caffeine, carbohydrate, fat, protein, and fiber

### Subgroup analysis

**Fig. 3:**
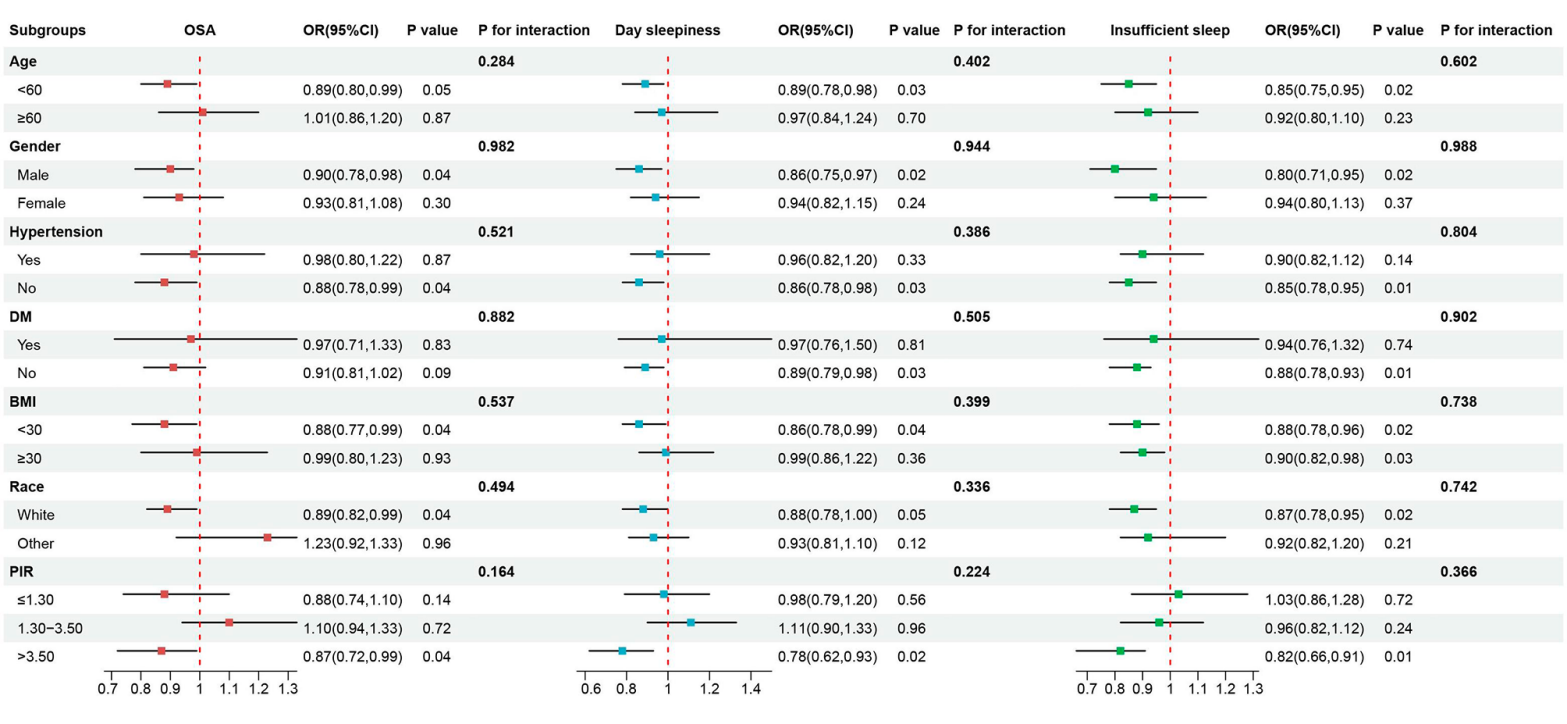
Subgroup analysis of the association between CDAI and three sleep-related outcomes

In order to examine whether the correlation between CDAI and each sleep-related outcome was robust in different population stratified by age, gender, hypertension, diabetes status, BMI, race and PIR, subgroup analyses were performed (Fig. 3). The results indicated that relatively stronger associations between CDAI and sleep outcomes were consistently found among young adults (age < 60 years), males, adults without hypertension and diabetes, the White and PIR > 3.50. No significant interactions of CDAI with these stratified variables were observed (all p for interaction > 0.050).

## Discussion

In this large-scale study of 7274 Americans from NHANES, we discovered that CDAI was non-linearly associated with lower risk of OSA and day sleepiness. At the same time, a linear inverse association between CDAI and insufficient sleep was observed. These findings highlighted the protective effect of antioxidative dietary and provided clinical evidence for the beneficial sleep health from increasing intake of dietary antioxidants.

To date, numerous epidemiological investigations have explored the efficacy of single antioxidant on sleep health, while few studies have focused on the effect of DTAC on sleep disorders. Kanagasabai et al. found that individuals with adequate sleep duration generally had an ideal level of serum vitamin C and other antioxidants profiles in comparison to short or very short sleeper [24]. Observational studies of vitamin A and sleep health are extremely limited and controversial. Natsuko et at. performed a questionnaire survey composed of 3304 Japanese female students and observed that the level of vitamin A intake was linked to disturbed wake–sleep cycle [25], whereas another study discovered there was no significant difference in serum vitamin A levels among adolescent girls with sleepiness comparing with control groups [26]. Although vitamin E was regarded as an important antioxidant, previous studies on vitamin E and sleep have also been controversial. Increased intake of vitamin E was associated with longer sleep time [27] and might reduce memory damage during chronic sleep deprivation [28]. In contrast, a randomized and stratified epidemiological study including 169 mild OSA patients and 83 moderate/severe OSA patients demonstrated that intake of vitamin A, C, D and E was not linked to OSA severity [29]. Interestingly, existing evidence from different observational research consistently supported that carotenoid consumption positively associated with sleep quality, which directly supported our findings in current study. For instance, Stringham et al. showed that healthy adults who were supplemented with 24 mg of macular carotenoid per day experienced improvements in sleep quality [30]. Furthermore, a NHANES 2005–2006 study also found that lower level of serum carotenoid concentration was associated with shorter sleep duration [31]. Despite all this, few studies have evaluated the interaction among different dietary antioxidants on sleep problems. A prior cross-sectional study of 265 diabetic women demonstrated that DTAC was inversely associated with poor sleep quality [11]. Similarly, another cross-sectional study conducted among Iranian post-menopausal women revealed that DTAC was inversely related to sleep disorders and other symptoms related menopause [12].

In present study, we utilized CDAI to comprehensively assess interactive effect of the antioxidants, which was an effective and promising strategy. Our results are generally reasonable. First, oxidative stress constitutes a critical hallmark of sleep disorders. A previous system review indicated that insufficient sleep could activate stress-related neurobiological mechanisms and trigger oxidative processes [32]. An increasing number of epidemiological evidence underlined the adverse effects of oxidative stress in patients with OSA, including the elevated level of intracellular reactive oxygen species (ROS) [33] and decreased level of nitric oxide (NO) or its derivatives [34, 35]. As a matter of fact, unlike other cells, the brain neurons are particularly vulnerable to oxidative stress since they tend to have higher metabolic demands and lower endogenous antioxidant levels [36]. Given that oxidative stress can aggravate impairment to the brain, contributing to the reduction of neurotransmitter concentrations and further affecting sleep quality [37], it is highly plausible that adequate antioxidant dietary may lower the risk of OSA and insufficient sleep via their powerful antioxidant properties.

Second, high-grade inflammation has been acted as a crucial role in the development of OSA [38, 39]. In fact, a prior meta-analysis has demonstrated that sleep disorders and insufficient sleep have elevated levels of c-reactive protein (CRP) and interleukin-6 (IL-6) [24, 40]. A study included three prospective US cohorts suggested that higher anti-inflammatory diet was related to a lower systemic inflammation among 8856 OSA participants [41]. So far, some researchers have verified a negative correlation between DTAC and serum CRP, involving the nuclear transcription factor-κB (NF-κB) pathway [37, 42]. Moreover, two Italian studies based on a two-week intervention with a high DTAC lead to lower CRP levels [43, 44]. Likewise, a population-based cohort study of 3853 Chinese women demonstrated that CDAI was inversely associated with levels of interleukin-1beta (IL-1β) and tumor necrosis factor-alpha (TNF-α) [45]. In addition, participants with higher CDAI in our study may consume more vitamins, vegetables and fruits and further display their protective benefits.

Third, glucose metabolism imbalance has been considered as a key risk factor of OSA as well as sleep disorders. A system review suggested that poorly controlled glucose levels could aggravate OSA by impacting central control of respiration and upper airway neural reflexes [37].To some extent, sufficient consumption of antioxidants has associated with lower glucose concentration [46] and improved pancreatic β-cell function [47]. A cohort study of 2694 older people aged more than 59 years old found that higher DTAC was linked to better glucose tolerance [48]. Similar results were found in younger [42] and middle-aged adults [49]. Furthermore, high intake of antioxidants from foods and beverages might provide dietary fiber, magnesium and some enzymes, which were important modifiers in glucose metabolism [50, 51]. Thus, a higher CDAI is related to more intake of these individual antioxidant nutrient, which have beneficial influence on our sleep health.

It is highlighted that non-linear associations between CDAI and the risk of OSA and day sleepiness were found in current study, suggesting the odds of OSA and day sleepiness decreased with the increase of CDAI in a certain range. However, the underlying mechanism of this non-linear relationship still remain unclear. Previous researches reported that dietary zinc and selenium had both properties of antioxidant and prooxidant, which could cause oxidative stress and lose antioxidant capacity when they were out of certain range [52, 53]. Dietary selenium has been linked to increased activation of white blood cells, proliferation and differentiation of immune cells, as well as the production of interferon γ (IFN γ) and other cytokines [54, 55]. Additionally, selenium exerts antioxidant, immunomodulatory and anti-inflammatory effects through the selenoproteins such as selenium-dependent glutathione peroxidase (GPx) [56]. However, an increase in GPx1 activity can potentially disrupt insulin function by excessively suppressing intracellular ROS required for insulin sensitization, ultimately leading to hyperglycemia [57]. Moreover, a meta-analysis of 5 randomized controlled trials has indicated that although selenium administration may improve insulin sensitivity to some extent, no positive effects on lipid profiles or glucose homeostasis have been observed [58]. Thirdly, it is noteworthy that a high dietary antioxidant index is not solely determined by an increased intake of dietary antioxidants, but is also influenced by the consumption of pro-oxidant foods. For instance, red meat, sugary foods, and starches can have pro-oxidant effects due to their high content of advanced glycation end products (AGEs), which are formed during cooking and processing [59]. AGEs can activate inflammatory pathways and generate ROS, which can damage cells and tissues in that counteract the positive effects of antioxidants on the body [60, 61]. These findings offer insight into the antioxidant paradox from various perspectives. Thus, it is possible that excessive intake of zinc and selenium may modify the protective effect of vitamins and carotenoids. More investigations are needed to confirm these dose-response relationship and understand these biological mechanisms.

### Strength and limitations

Our study has several noteworthy advantages. First of all, our study data is based on NHANES, which provides a large-scale and nationally representative database, making our study findings reliable. Second, we further determine a promising and comprehensive index for OSA patients by dietary intervention, which fully consider the potential interactions between different antioxidant nutrients. However, some limitations should be noted. First, information about dietary intake was obtained only at baseline without dynamic follow-up. Moreover, two 24-hour recall dietary interviews are limitations regarding correlations between antioxidants and sleep disorders because blood levels of antioxidants would be preferable. Second, the diagnosis of OSA was used by face-to-face interview but not respiratory sleep monitoring, which was a gold standard in clinical practice. Third, given the nature of cross-sectional design, these causal associations cannot be ensured, so more prospective and well-designed studies are urgent to conduct in the future. Finally, although some conventional variables were fully controlled in current study, other measured confounders were not considered.

## Conclusion

In summary, the present research indicated that CDAI was non-linearly associated with lower risk of OSA and day sleepiness whereas a linear inverse association between CDAI and insufficient sleep was observed. These findings implicated that combined intake of antioxidants could be a promising and effective approach to optimize sleep quality for public. Further large-scale research is urgent to demonstrate these associations and biological mechanisms.

## Data Availability

Detailed information about this study can be found at NHANES online website: https://www.cdc.gov/nchs/nhanes/index.htm.

## Abbreviations

PIR: poverty income ratio
BMI: body mass index
CVD: cardiovascular disease
OSA: Obstructive sleep apnea
OR: odds ratio
CI: confidence interval
RCS: restricted cubic spline
IFNγ: interferonγ
GPx: glutathione peroxidase
AGEs: advanced glycation end products

## References

[1] Deng M-G, Nie J-Q, Li Y-Y, Yu X, Zhang Z-J, Higher (2022). HEI-2015 scores are Associated with Lower Risk of Sleep Disorder: results from a nationally Representative Survey of United States adults. Nutrients.

[2] Zhao M, Tuo H, Wang S, Zhao L (2020). The effects of Dietary Nutrition on Sleep and Sleep disorders. Mediators Inflamm.

[3] Rotenberg BW, Murariu D, Pang KP (2016). Trends in CPAP adherence over twenty years of data collection: a flattened curve. J Otolaryngol Head Neck Surg.

[4] Weaver TE, Grunstein RR (2008). Adherence to continuous positive airway pressure therapy: the challenge to effective treatment. Proc Am Thorac Soc.

[5] Zheng Y, Zhang L, Bonfili L, de Vivo L, Eleuteri AM, Bellesi M (2023). Probiotics Supplementation attenuates inflammation and oxidative stress Induced by Chronic Sleep Restriction. Nutrients.

[6] Gasmi A, Semenova Y, Noor S, Benahmed AG, Bjørklund G. Sleep, dietary melatonin supplementation, and COVID-19. Curr Med Chem. 2023;31:1298–314. 10.2174/0929867330666230224093849 36825700

[7] Ahmad SB, Ali A, Bilal M, Rashid SM, Wani AB, Bhat RR et al. Melatonin and health: insights of Melatonin Action, Biological functions, and Associated disorders. Cell Mol Neurobiol. 2023;:1–22. 10.1007/s10571-023-01324-w PMC9907215 36752886

[8] Pak VM, Lee J (2022). Examining the role of micronutrients on improving long COVID sleep-related symptoms. J Clin Nurs.

[9] Sheng L-T, Jiang Y-W, Pan A, Koh W-P (2022). Dietary total antioxidant capacity and mortality outcomes: the Singapore Chinese Health Study. Eur J Nutr.

[10] Bjelakovic G, Nikolova D, Gluud LL, Simonetti RG, Gluud C (2007). Mortality in randomized trials of antioxidant supplements for primary and secondary prevention: systematic review and meta-analysis. JAMA.

[11] Daneshzad E, Keshavarz S-A, Qorbani M, Larijani B, Azadbakht L (2020). Dietary total antioxidant capacity and its association with sleep, stress, anxiety, and depression score: a cross-sectional study among diabetic women. Clin Nutr ESPEN.

[12] Abshirini M, Siassi F, Koohdani F, Qorbani M, Khosravi S, Hedayati M (2018). Dietary total antioxidant capacity is inversely related to menopausal symptoms: a cross-sectional study among Iranian postmenopausal women. Nutrition.

[13] Zipf G, Chiappa M, Porter KS, Ostchega Y, Lewis BG, Dostal J (2013). National health and nutrition examination survey: plan and operations, 1999–2010. Vital Health Stat.

[14] Borrud L, Chiappa MM, Burt VL, Gahche J, Zipf G, Johnson CL et al. National Health and Nutrition Examination Survey: national youth fitness survey plan, operations, and analysis, 2012. Vital Health Stat 2. 2014;:1–24. 24709592

[15] NHANES 2005–2006.: Sleep Disorders Data Documentation, Codebook, and Frequencies. https://wwwn.cdc.gov/Nchs/Nhanes/2005-2006/SLQ_D.htm#SLQ030. Accessed 28 Dec 2023.

[16] Chaput J-P, Dutil C, Sampasa-Kanyinga H (2018). Sleeping hours: what is the ideal number and how does age impact this?. Nat Sci Sleep.

[17] Hirshkowitz M, Whiton K, Albert SM, Alessi C, Bruni O, DonCarlos L (2015). National Sleep Foundation’s sleep time duration recommendations: methodology and results summary. Sleep Health.

[18] Scinicariello F, Buser MC, Feroe AG, Attanasio R (2017). Antimony and sleep-related disorders: NHANES 2005–2008. Environ Res.

[19] Zhang HR, Yang Y, Tian W, Sun YJ (2022). Dietary Fiber and all-cause and Cardiovascular Mortality in older adults with hypertension: a cohort study of NHANES. J Nutr Health Aging.

[20] Maugeri A, Hruskova J, Jakubik J, Kunzova S, Sochor O, Barchitta M (2019). Dietary antioxidant intake decreases carotid intima media thickness in women but not in men: a cross-sectional assessment in the Kardiovize study. Free Radic Biol Med.

[21] Rattan P, Penrice DD, Ahn JC, Ferrer A, Patnaik M, Shah VH (2022). Inverse Association of Telomere length with Liver Disease and Mortality in the US Population. Hepatol Commun.

[22] Lloyd-Jones DM, Ning H, Labarthe D, Brewer L, Sharma G, Rosamond W (2022). Status of Cardiovascular Health in US adults and Children Using the American Heart Association’s New Life’s essential 8 Metrics: Prevalence Estimates from the National Health and Nutrition Examination Survey (NHANES), 2013 through 2018. Circulation.

[23] Willett WC, Howe GR, Kushi LH. Adjustment for total energy intake in epidemiologic studies. Am J Clin Nutr. 1997;65 4 Suppl:1220S-1228S; discussion 1229S-1231S. 10.1093/ajcn/65.4.1220S 9094926

[24] Kanagasabai T, Ardern CI (2015). Contribution of inflammation, oxidative stress, and antioxidants to the relationship between Sleep Duration and Cardiometabolic Health. Sleep.

[25] Sato-Mito N, Sasaki S, Murakami K, Okubo H, Takahashi Y, Shibata S (2011). The midpoint of sleep is associated with dietary intake and dietary behavior among young Japanese women. Sleep Med.

[26] Bahrami A, Khorasanchi Z, Sadeghnia HR, Tayefi M, Avan A, Ferns GA (2019). Depression in adolescent girls: relationship to serum vitamins a and E, immune response to heat shock protein 27 and systemic inflammation. J Affect Disord.

[27] Grandner MA, Kripke DF, Naidoo N, Langer RD (2010). Relationships among dietary nutrients and subjective sleep, objective sleep, and napping in women. Sleep Med.

[28] Alzoubi KH, Khabour OF, Rashid BA, Damaj IM, Salah HA (2012). The neuroprotective effect of vitamin E on chronic sleep deprivation-induced memory impairment: the role of oxidative stress. Behav Brain Res.

[29] Chrysostomou S, Frangopoulos F, Koutras Y, Andreou K, Socratous L, Giannakou K (2022). The relation of dietary components with severity of obstructive sleep apnea in Cypriot patients: a randomized, stratified epidemiological study. PLoS ONE.

[30] Stringham JM, Stringham NT, O’Brien KJ (2017). Macular Carotenoid Supplementation Improves Visual Performance, Sleep Quality, and adverse physical symptoms in those with high screen time exposure. Foods.

[31] Beydoun MA, Gamaldo AA, Canas JA, Beydoun HA, Shah MT, McNeely JM (2014). Serum nutritional biomarkers and their associations with sleep among US adults in recent national surveys. PLoS ONE.

[32] Villafuerte G, Miguel-Puga A, Rodríguez EM, Machado S, Manjarrez E, Arias-Carrión O (2015). Sleep deprivation and oxidative stress in animal models: a systematic review. Oxid Med Cell Longev.

[33] Dyugovskaya L, Lavie P, Lavie L (2002). Increased adhesion molecules expression and production of reactive oxygen species in leukocytes of sleep apnea patients. Am J Respir Crit Care Med.

[34] Schulz R, Schmidt D, Blum A, Lopes-Ribeiro X, Lücke C, Mayer K (2000). Decreased plasma levels of nitric oxide derivatives in obstructive sleep apnoea: response to CPAP therapy. Thorax.

[35] Ip MS, Lam B, Chan LY, Zheng L, Tsang KW, Fung PC (2000). Circulating nitric oxide is suppressed in obstructive sleep apnea and is reversed by nasal continuous positive airway pressure. Am J Respir Crit Care Med.

[36] Lehtinen MK, Bonni A (2006). Modeling oxidative stress in the central nervous system. Curr Mol Med.

[37] Reutrakul S, Mokhlesi B (2017). Obstructive sleep apnea and diabetes: a state of the Art Review. Chest.

[38] Dempsey JA, Veasey SC, Morgan BJ, O’Donnell CP (2010). Pathophysiology of sleep apnea. Physiol Rev.

[39] Unnikrishnan D, Jun J, Polotsky V (2015). Inflammation in sleep apnea: an update. Rev Endocr Metab Disord.

[40] Irwin MR, Olmstead R, Carroll JE (2016). Sleep disturbance, Sleep Duration, and inflammation: a systematic review and Meta-analysis of Cohort studies and experimental sleep deprivation. Biol Psychiatry.

[41] Liu Y, Tabung FK, Stampfer MJ, Redline S, Huang T (2022). Overall diet quality and proinflammatory diet in relation to risk of obstructive sleep apnea in 3 prospective US cohorts. Am J Clin Nutr.

[42] Hermsdorff HHM, Puchau B, Volp ACP, Barbosa KB, Bressan J, Zulet MÁ (2011). Dietary total antioxidant capacity is inversely related to central adiposity as well as to metabolic and oxidative stress markers in healthy young adults. Nutr Metab (Lond).

[43] Valtueña S, Pellegrini N, Franzini L, Bianchi MA, Ardigò D, Del Rio D (2008). Food selection based on total antioxidant capacity can modify antioxidant intake, systemic inflammation, and liver function without altering markers of oxidative stress. Am J Clin Nutr.

[44] Franzini L, Ardigò D, Valtueña S, Pellegrini N, Del Rio D, Bianchi MA (2012). Food selection based on high total antioxidant capacity improves endothelial function in a low cardiovascular risk population. Nutr Metab Cardiovasc Dis.

[45] Luu HN, Wen W, Li H, Dai Q, Yang G, Cai Q (2015). Are dietary antioxidant intake indices correlated to oxidative stress and inflammatory marker levels?. Antioxid Redox Signal.

[46] Yao LH, Jiang YM, Shi J, Tomás-Barberán FA, Datta N, Singanusong R (2004). Flavonoids in food and their health benefits. Plant Foods Hum Nutr.

[47] Sotoudeh G, Abshirini M, Bagheri F, Siassi F, Koohdani F, Aslany Z (2018). Higher dietary total antioxidant capacity is inversely related to prediabetes: a case-control study. Nutrition.

[48] Okubo H, Syddall HE, Phillips DIW, Sayer AA, Dennison EM, Cooper C (2014). Dietary total antioxidant capacity is related to glucose tolerance in older people: the Hertfordshire Cohort Study. Nutr Metab Cardiovasc Dis.

[49] Psaltopoulou T, Panagiotakos DB, Pitsavos C, Chrysochoou C, Detopoulou P, Skoumas J (2011). Dietary antioxidant capacity is inversely associated with diabetes biomarkers: the ATTICA study. Nutr Metab Cardiovasc Dis.

[50] Ford ES, Mokdad AH (2001). Fruit and vegetable consumption and diabetes mellitus incidence among U.S. adults. Prev Med.

[51] Valko M, Leibfritz D, Moncol J, Cronin MTD, Mazur M, Telser J (2007). Free radicals and antioxidants in normal physiological functions and human disease. Int J Biochem Cell Biol.

[52] Lee KH, Jeong D (2012). Bimodal actions of selenium essential for antioxidant and toxic pro-oxidant activities: the selenium paradox (review). Mol Med Rep.

[53] Yuan Y, Niu F, Liu Y, Lu N (2014). Zinc and its effects on oxidative stress in Alzheimer’s disease. Neurol Sci.

[54] Buonacera A, Stancanelli B, Colaci M, Malatino L (2022). Neutrophil to lymphocyte ratio: an emerging marker of the relationships between the Immune System and diseases. Int J Mol Sci.

[55] Bentley-Hewitt KL, Chen RK-Y, Lill RE, Hedderley DI, Herath TD, Matich AJ (2014). Consumption of selenium-enriched broccoli increases cytokine production in human peripheral blood mononuclear cells stimulated ex vivo, a preliminary human intervention study. Mol Nutr Food Res.

[56] Rotruck JT, Pope AL, Ganther HE, Swanson AB, Hafeman DG, Hoekstra WG (1973). Selenium: biochemical role as a component of glutathione peroxidase. Science.

[57] McClung JP, Roneker CA, Mu W, Lisk DJ, Langlais P, Liu F (2004). Development of insulin resistance and obesity in mice overexpressing cellular glutathione peroxidase. Proc Natl Acad Sci U S A.

[58] Tabrizi R, Akbari M, Moosazadeh M, Lankarani KB, Heydari ST, Kolahdooz F (2017). The effects of Selenium supplementation on glucose metabolism and lipid profiles among patients with metabolic diseases: a systematic review and Meta-analysis of Randomized controlled trials. Horm Metab Res.

[59] Nowotny K, Schröter D, Schreiner M, Grune T (2018). Dietary advanced glycation end products and their relevance for human health. Ageing Res Rev.

[60] Uribarri J, Cai W, Peppa M, Goodman S, Ferrucci L, Striker G (2007). Circulating glycotoxins and dietary advanced glycation endproducts: two links to inflammatory response, oxidative stress, and aging. J Gerontol Biol Sci Med Sci.

[61] Byun K, Yoo Y, Son M, Lee J, Jeong G-B, Park YM (2017). Advanced glycation end-products produced systemically and by macrophages: a common contributor to inflammation and degenerative diseases. Pharmacol Ther.

